# Flexible Doppler ultrasound device for the monitoring of blood flow velocity

**DOI:** 10.1126/sciadv.abi9283

**Published:** 2021-10-27

**Authors:** Fengle Wang, Peng Jin, Yunlu Feng, Ji Fu, Peng Wang, Xin Liu, Yingchao Zhang, Yinji Ma, Yingyun Yang, Aiming Yang, Xue Feng

**Affiliations:** 1 AML, Department of Engineering Mechanics, Tsinghua University, Beijing 100084, China.; 2 Center for Flexible Electronics Technology, Tsinghua University, Beijing 100084, China.; 3 Department of Gastroenterology, Peking Union Medical College Hospital, Beijing 100730, China.; 4 Institute of Flexible Electronics Technology of THU, Jiaxing 314000, China.

## Abstract

Thrombosis and restenosis after vascular reconstruction procedures may cause complications such as stroke, but a clinical means to continuously monitor vascular conditions is lacking. Conventional ultrasound probes are rigid, particularly for postoperative patients with fragile skin. Techniques based on photoplethysmography or thermal analysis provide only relative changes in flow volume and have a shallow detection depth. Here, we introduce a flexible Doppler ultrasound device for the continuous monitoring of the absolute velocity of blood flow in deeply embedded arteries based on the Doppler effect. The device is thin (1 mm), lightweight (0.75 g), and skin conforming. When the dual-beam Doppler method is used, the influence of the Doppler angle on the velocity measurement is avoided. Experimental studies on ultrasound phantoms and human subjects demonstrate accurate measurement of the flow velocity. The wearable Doppler device has the potential to enhance the quality of care of patients after reconstruction surgery.

## Teaser

Flexible, angled transducer arrays offer direct blood flow measurements.

## INTRODUCTION

The variation in blood flow in time and space contains abundant information regarding a person’s cardiovascular situation. Blood flow in arteries varies in a pulsatile manner because of intermittent heart pumping, and the flow velocity near the blood vessel wall is lower than that in the center of a vessel. The characteristics of vessels from different regions, such as their dimensions and compliance, have an impact on the hemodynamics occurring within them (*1*, *2*). Flow velocity parameters can indicate thrombus, artery stenosis, hardening, occlusion, and other diseases (*3*–*6*). Long-term continuous monitoring of the blood flow velocity may be valuable in the diagnosis and prognosis of some vascular conditions (*7*, *8*). For instance, ischemic stroke occurs when an artery to the brain becomes blocked, which is partly caused by narrowing of the carotid artery (stenosis) and the formation of blood clots that can block the carotid artery (thrombosis) or can break off and travel to the brain through the bloodstream (embolism). Carotid endarterectomy is an accepted and effective procedure for preventing strokes due to carotid artery stenosis (*9*), but acute thrombosis may occur at the endarterectomy and clamping sites within 72 hours, causing postoperative stroke and increasing postoperative morbidity and mortality (*10*, *11*). Restenosis may occur even within 1 year because of intimal hyperplasia or the progression of an underlying atherosclerotic disease after surgery (*9*, *12*). Prompt recognition and immediate reoperation are paramount (*9*, *10*). However, patients are often examined only if ischemic symptoms recur (*13*), making immediate reoperation necessary. Postoperative thrombosis can also occur after other vascular reconstruction procedures, including angioplasty and stenting, and replantation operations. To prevent thrombosis at the site of vessel anastomosis after replantation surgery, a common assessment is a physical exam of the capillary fill and skin color every hour (*14*, *15*). Patients can receive only a limited number of assessments when they are in the hospital and are sporadically or even not assessed after discharge, missing the opportunity to save their tissue/grafts (*16*). Therefore, continuous monitoring of the blood flow velocity of patients and evaluation of these conditions is necessary in clinical practice and health care settings.

At present, the common clinical way to measure blood flow is to use ultrasound equipment, including the handheld ultrasound device, which integrates the B-mode and Doppler function with rigid probes, resulting in high requirements on the handhold stability for the operator and possible compression to the local vessels. Implanted Doppler probes can achieve continuous monitoring but are limited by wired connection requirements (*17*–*19*). Other blood flow monitoring technologies have been proposed in recent years based on photoplethysmography (PPG) (*20*), thermal analysis (*7*, *21*), and fringe-field capacitor technology (*16*). PPG and the fringe-field capacitive sensor provide only an estimate of changes in blood volume, and thermal analysis provides only a relative time-averaged value of the flow velocity. All three of these technologies are limited to measuring depths of only a few millimeters. Compared with light and heat, ultrasound has a higher penetration capability and causes no harm to the human body within a safe intensity (*22*). The ultrasound Doppler technique is the only method that can feed rapid changes in cardiovascular conditions back in real time, including the absolute mean and peak velocity of blood flow. In addition, to detect small-vessel lumen reductions, the measurement of velocity changes is more sensitive than the measurement of flow (*13*). Flexible electronic devices are soft and wearable and have been proven to have the ability to monitor various vital signs continuously (*23*–*29*). In this work, we make use of the powerful features of ultrasound techniques and flexible electronics to reduce possible damage to patients.

Here, we present a flexible continuous-wave (CW) Doppler ultrasound device for the real-time, continuous monitoring of absolute blood flow velocity without averaging. The piezoelectric transducers are inclined to have a certain angle with the skin surface to emit oblique ultrasound beams to produce the Doppler effect. The soft and thin structure of the device ensures conformal light contact with the curved skin surface. The array design and the use of the dual-beam ultrasound Doppler (DBUD) method avoid the influence of the Doppler angle on the velocity measurement and allow for obtaining absolute velocity, and thus eliminate the need for calibration. The device is demonstrated first by an ultrasound phantom and then on human arteries, showing its excellent ability to monitor blood flow velocity.

## RESULTS

### Working principle and device design

**Fig. 1.**
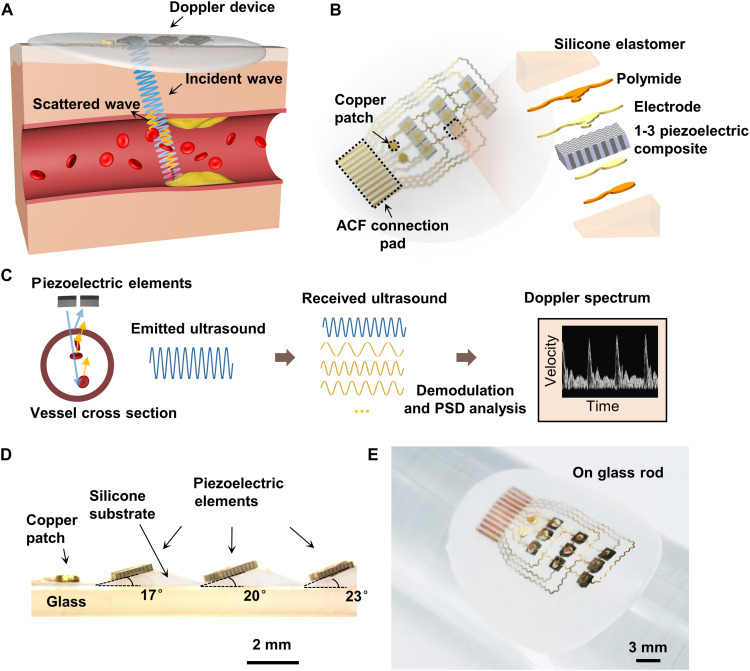
Schematic diagram of the principle and design of the Doppler ultrasonic device. (**A**) Schematic of the Doppler ultrasonic device. The device continuously transmits ultrasound waves and receives echoes from a moving scatterer (such as red blood cells). (**B**) Schematics (left) and exploded view (right) of the device structure. ACF, Anisotropic Conductive Film. (**C**) Detection concept. Each received echo is Doppler-shifted relative to the frequency transmitted by the transducer, which is related to the velocity of the scatterer. A mixture of Doppler frequency shifts that changes from moment to moment and from place to place within the vessel lumen is produced by many moving scatterers in blood flow. After demodulation and power spectral density (PSD) analysis, the Doppler spectrum that contains the absolute frequency shift or velocity is obtained. (**D**) Optical image of the cross section of the semifinished device before top electrode bonding. The device with transducer arrays, bottom electrodes, and substrate was placed on a glass plate. (**E**) Optical image of the device when bent around a curved surface. Photo credit: Fengle Wang, Tsinghua University.

Ultrasonic measurements of blood flow velocity are based on the Doppler effect. Piezoelectric transducers transmit ultrasound waves with a frequency of *f*_0_ into the skin. When an echo comes from a moving scatterer (such as red blood cells), the received frequency *f*_R_ has a certain deviation from the transmitted frequency, that is, a Doppler shift *f*_D_

$$f_{D}=f_{R}-f_{0}=\frac{2f_{0}V\text{cos}\;\theta }{c}$$

where *c* is the speed of sound, *V* is the flow velocity, and θ, known as the Doppler angle, is the angle between the axis of the ultrasound beam and the direction of flow, looking toward the transducer (Fig. 1A).

When the scatterer moves relative to the probe (θ is not equal to 90°), the received echo will exhibit a certain frequency shift. If the scatterer moves toward the probe (0 ≤ θ < 90°), the echo frequency will be higher than the transmitted frequency, which is called “forward”; if the scatterer moves away from the probe (90° < θ ≤ 180°), the echo frequency will be lower than the transmitted frequency, which is called “reverse.”

The Doppler signal of blood flow is a mixture of many single-frequency signals of scatterers with different velocities in the blood flow. Each of these has a particular amplitude, frequency, and phase (Fig. 1C). The actual received signal needs to be demodulated before extracting Doppler signals (*1*, *30*). Relatively low-frequency Doppler signals from slowly moving vessel walls and muscular tissue will be eliminated from the output by applying a high-pass filter (*1*, *31*). The power spectrum is computed by spectral analysis to display the relative contribution of each frequency component to the original signal along a vertical line. The height represents a frequency bin, which can be converted into a velocity value, and the brightness represents the signal power or intensity for the bin, which is also the relative number of scatterers with the corresponding velocity (the rightmost image of Fig. 1C). The detailed signal processing procedure is described in note S1.

CW Doppler is used in our device. One transducer continuously transmits an ultrasound beam, and another adjacent transducer receives the scattered echoes from the blood. Alternatively, one middle transducer receives and two adjacent transducers transmit simultaneously to reinforce sound intensity for a larger depth of penetration. CW Doppler offers simple signal processing, easy operation, and an unlimited maximum measurable flow velocity compared with pulse-wave Doppler (further discussions are provided in note S2) (*1*, *30*). Flow velocities in small vessels like capillaries are very low, which have little impact on the monitoring of carotid, branchial, and other arteries.

To design a flexible Doppler ultrasound device, the key problems are producing ultrasonic Doppler effects and determination of the Doppler angle θ. To solve the first problem, we used the angled transducer array to generate oblique ultrasound beams so that there is relative motion between the ultrasound beams and the scatterers. The second problem is solved by DBUD method (see the “DBUD method” section). On the basis of this principle, we designed a flexible Doppler ultrasonic device as shown in Fig. 1B. The device consists of a 3 by 3 array of oblique 1-3 composite piezoelectric transducers, flexible circuits, and a soft silicone package. With a working frequency of 5 MHz, the piezoelectric transducer is 1.5 mm by 1.5 mm in size and has a certain inclination angle to realize oblique incidence of the ultrasound beam and produce the Doppler effect with the scatterers in the moving blood flow (Fig. 1D). The design avoids the phase control problem of phased array technology because of the curved skin surface (a further discussion is provided in note S2). The working frequency of 5 MHz ensures sufficient intensity of ultrasonic waves scattered from the carotid blood flow and avoids significant attenuation losses (see more in note S4). The 1-3 composite has low acoustic impedance (15 megarayleigh) and thus can achieve better acoustic impedance matching with the silicone substrate and human skin. Each transducer contacts the top common ground electrode and the bottom exclusive simulating electrode. The top ground and bottom electrodes are routed to the same plane through a copper patch. The multielement design (0.3 mm apart from each other) in each row helps cover the blood vessel below, ensuring that ultrasound beams pass through blood flow and avoiding high operation requirements. The overall thickness is only 1.04 mm, and the weight is approximately 0.75 g. Ecoflex silicone has an ultralow modulus of only approximately 5 kPa (*23*). The fabrication process of the device is based on photoetching technology and dry etching, which ensure the patterning of Cu/polyimide (PI) electrode on the silicone substrates with slopes (see Methods and fig. S2). The device can be attached to a curved surface and bear large deformation (Fig. 1E).

### Device characterization

**Fig. 2.**
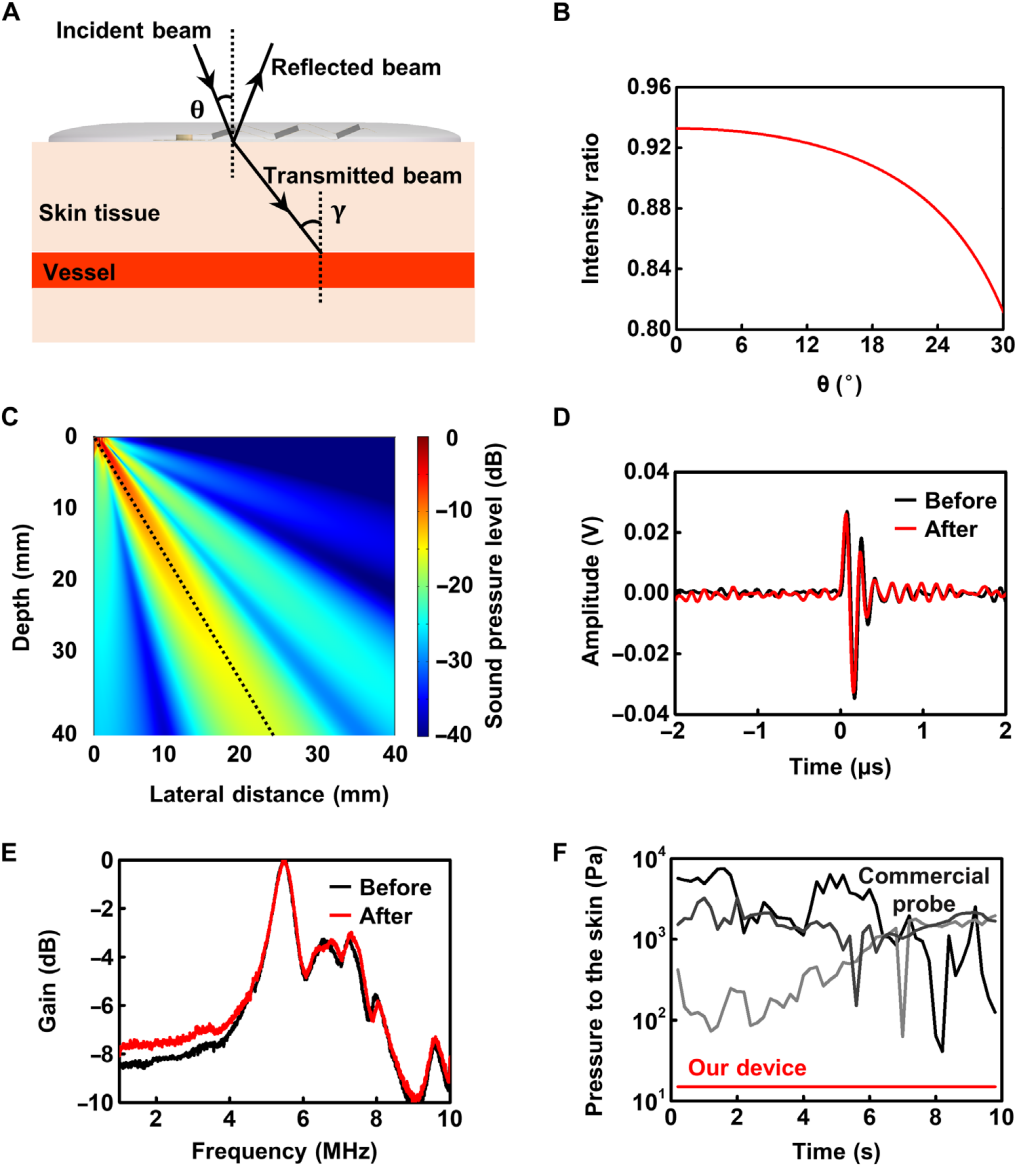
Device characterization and performance. (**A**) Schematic of oblique incidence of the ultrasound beam through the Ecoflex substrate and skin. The ultrasound beam will refract on the interface because of acoustic impedance mismatch. (**B**) Relationship between the intensity ratio of the transmitted wave to the incident wave and the inclination angle of the transducer θ. (**C**) Simulated beam pattern of a piezoelectric transducer with a 20° inclination angle. (**D** and **E**) Impulse response (A) and frequency response (B) of the transducer before and after being bent 500 times. (**F**) Comparison of the amount of pressure applied to the skin during measurements. The dark, dark gray, and light gray lines represent three measurements of variations in the pressure with time produced by the commercial probe. The red line represents the results of our Doppler device.

Piezoelectric transducers with different inclination angles are used to avoid adjusting the angle for various parts of the human body, such as the traditional ultrasound probe examination, and to overcome the influence of the Doppler angle on velocity measurement by using the DBUD method (see the “DBUD method” section). When considering the determination of the inclination angle θ, note first that due to the acoustic impedance mismatch between the Ecoflex substrate and skin, ultrasound will refract on the interface; thus, the angle of the ultrasonic beam injected into the vessel γ is greater than θ (Fig. 2A). The incident wave will lose part of the energy because of reflection, and the intensity ratio of the transmitted wave to the incident wave *I_t_*/*I_i_* is given by the following formula

$$\frac{I_{t}}{I_{i}}=\frac{4Z_{1}Z_{2}{\text{cos}}^{2}\theta }{{\left(Z_{1}\text{cos}\;\gamma +Z_{2}\text{cos}\;\theta \right)}^{2}}$$

where *Z*_1_ and *Z*_2_ refer to the acoustic impedances of the Ecoflex and skin, respectively. The larger is the inclination angle, the greater is the energy loss of the incident wave (Fig. 2B). On the other hand, the smaller is the inclination angle, the smaller is the Doppler frequency shift. To obtain the appropriate frequency shift and avoid significant refraction energy loss, we set θ at 17°, 20°, and 23° in the three rows of transducers, with refraction angles of 26.76°, 31.78°, and 36.99°, respectively (the sound velocities of human tissue and Ecoflex are set to 1540 and 1000 m/s, respectively). If the transducer arrays contain more rows, the range of inclination angle can be set larger to fit various arteries. The beam pattern of the transducer with a 20° inclination angle is calculated using finite element analysis software (COMSOL Multiphysics 5.0). The results show that the transducer has excellent beam directivity (Fig. 2C).

Stable performance is very important for flexible ultrasound devices attached to the deformable skin surface. We measured the pulse response and frequency response of the transducer before and after being bent 500 times (Fig. 2, D and E). The results show that its performance remains unchanged before and after bending, and the central frequency is stable at approximately 5 MHz. In addition, the device does not need to be pressed by hand to maintain stability during operation. Because of its extremely low weight, the pressure on the skin is much smaller than that of the traditional probe. Figure 2F compares the pressure produced by the device attached to the skin with that produced by a traditional probe (5-MHz double-crystal Doppler probe; Lanhui Chaosheng Company). Our device applies minimal pressure (approximately 15 Pa) due to its light weight, while the pressure from the traditional probe can exceed 1000 Pa, and its variance is considerably large. This results in discomfort and inaccurate recordings when the traditional probe is used for monitoring over long periods and may cause damage especially for postoperative patients with fragile skin.

### In vitro device characterization

**Fig. 3.**
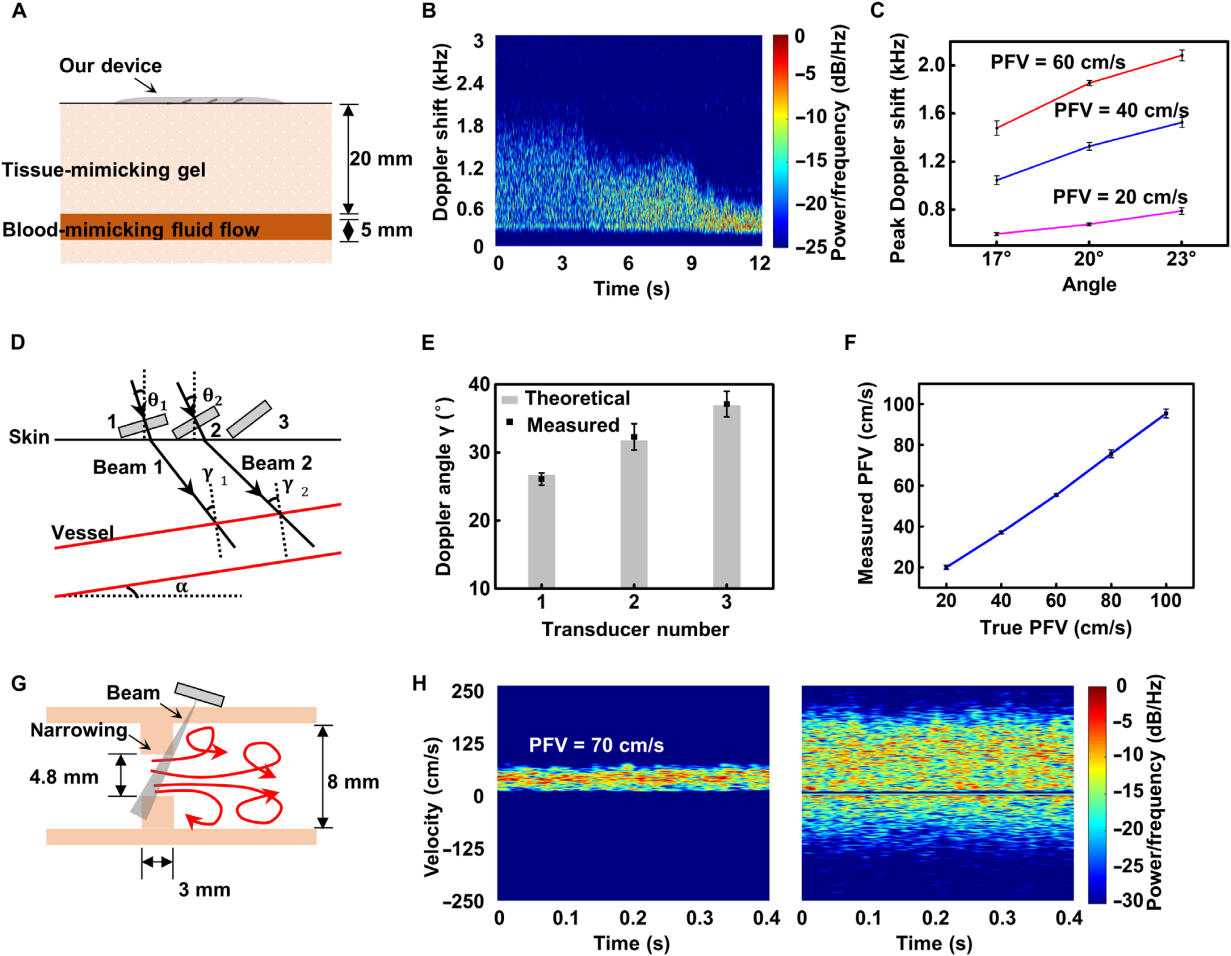
In vitro device validation. (**A**) Structure specification of ultrasound phantom. (**B**) Doppler spectrum of blood-mimicking flow with PFVs varying from 60 to 20 cm/s by using 17° transducers. The time is represented on the horizontal axis of the Doppler spectrum at the base of the display. The frequency bin is shown on the vertical axis of the spectrum, which can be converted into a velocity value using the Doppler equation. Both the forward and reverse Doppler signals are displayed simultaneously on the same spectrum, with flow toward the transducer displayed on the positive half-axis and flow away from the transducer displayed on the negative half-axis. The distribution of frequency bins within the sample volume is illustrated by the brightness of the spectral display. The brightness of a pixel is proportional to the number of scatterers causing that frequency shift at that specific point in time. (**C**) Measurements by 17°, 20°, and 23° transducers at three PFVs of 20, 40, and 60 cm/s. (**D**) Schematic of the DBUD method. (**E**) Calculations of the Doppler angle γ by using the pairwise results of the Doppler peak shifts produced by the 17° (1), 20° (2), and 23° (3) transducers. Error bars represent ±SD (*N* = 5). (**F**) Measured PFV versus true PFV curves. Error bars represent ±SD (*N* = 5). (**G**) Schematic of the flow through a constriction followed by a rapid expansion downstream, showing the regions of turbulence. (**H**) Spectra of flow in the normal segment of the tube (left) and flow around the narrowing site (right). The velocity increases as the blood flows through the narrowing site (from left to right) followed by an area of flow reversal beyond the narrowing.

The device was first tested on a standard ultrasound phantom. The ultrasonic phantom is a testing device consisting of tissue-mimicking material and blood-mimicking fluid that simulates the acoustic characteristics of real human tissues. The ultrasound phantom that we used was Optimizer 1425A (Gammex Inc.), which has a variable flow system and a 5-mm-diameter vessel placed horizontally at a depth of 20 mm (Fig. 3A). Figure 3B shows the Doppler spectrum of the blood-mimicking flow under different velocities. Within 12 s, we adjusted the peak flow velocity (PFV) to 60, 40, and 20 cm/s successively. In general, the flow velocity near the vessel wall is slower than that near the center because of friction; thus, the flow produces a mixture of large and small Doppler frequency shifts. As Fig. 3B shows, from 60 to 20 cm/s, the peak Doppler frequency shift decreased, the range of the frequency shift was reduced, and the intensity of the Doppler signal increased. These results indicate that when the velocity range of scatterers decreases, the number of scatterers with similar velocities increases. We calculated the peak Doppler frequency shifts produced by transducers with three inclination angles at three velocities by using the modified geometric method (*32*). With increasing inclination angle and velocity, the peak frequency shift rises, which can also be concluded from the Doppler equation (Fig. 3C).

### DBUD method

The ultrasound Doppler effect can accurately measure the blood flow velocity, but the angle between the direction of flow and the ultrasound beam must be known. The problem is that the angle between the direction of the vessel and skin surface is uncertain. A commercial ultrasound machine that can carry out B-mode ultrasound imaging can calculate the Doppler angle from an image. Taking advantage of piezoelectric transducer arrays with different inclination angles, we can overcome the influence of the Doppler angle on the velocity measurements to quantitatively measure the flow velocity. The principle of this method, which is called DBUD, is shown in Fig. 3D. Two beams with different angles of incidence, θ_1_ and θ_2_, are emitted by CW transducers. According to the Doppler effect and geometrical conditions, we obtained the following equations

$$\left\{ \begin{array}{c} f_{D1}=\frac{2v\;\text{sin}\;\gamma_{1}}{c}f_{0}\\ f_{D2}=\frac{2v\;\text{sin}\;\gamma_{2}}{c}f_{0}\\ {\text{sin}}^{-1}(\text{sin}\frac{\theta_{1}}{\eta })-{\text{sin}}^{-1}(\text{sin}\frac{\theta_{2}}{\eta })=\gamma_{1}-\gamma_{2} \end{array}\right.$$

where η is the refractive index, *c* is the sound velocity in blood, and γ_1_ and γ_2_ are the Doppler angles. From these equations, a unique solution of Doppler angles, flow velocity *v*, and inclination angle of vessel α can be derived (see note S3).

Through pairwise calculations of the previous Doppler peak shifts produced by the 17°, 20°, and 23° transducers, the obtained Doppler angles are close to theoretical values (Fig. 3E). Using the calculated Doppler angles, we measured the blood-mimicking flow under different PFVs from 20 to 100 cm/s. The results show good agreement with the actual PFV, and the SD of the PFV is within 2% (Fig. 3F). The relative error of PFV is below 8.69%. The method can effectively calculate the Doppler angle and measure the absolute flow velocity without calibration.

### In vitro narrowing experiment

Atherosclerosis or a thrombus can cause artery narrowing (stenosis) (*33*). Narrowing leads to increased resistance to blood flow, and excessive narrowing even completely occludes the artery. Blood generally flows through arteries in a laminar flow pattern, namely, the movement of blood in parallel lines. However, the velocity increases suddenly as the blood encounters a stenosis, causing turbulence beyond the narrowing (*13*). An in vitro narrowing simulator was developed, as shown in Fig. 3G. The blood-mimicking fluid flows at a constant speed in a silicone tube with an inner diameter of 8 mm. A 3-mm-long segment was cut from a silicone tube with an outer diameter of 8 mm and an inner diameter of 4.8 mm and was then inserted into the former tube to simulate narrowing. The left side of Fig. 3H shows the spectrum of flow in the normal segment of the tube, where the blood flow is relatively regular. Only positive flow occurs (PFV, 70 cm/s), and the flow velocity is relatively consistent. The figure on the right is the spectrum of flow around the narrowing site. The spectrum of velocity is obviously broadened, with an unstable peak velocity (>180 cm/s) and reverse flow, which are the characteristics of turbulence. Therefore, our device can capture the flow characteristics of the fluid under different conditions.

### Blood flow velocity monitoring

**Fig. 4.**
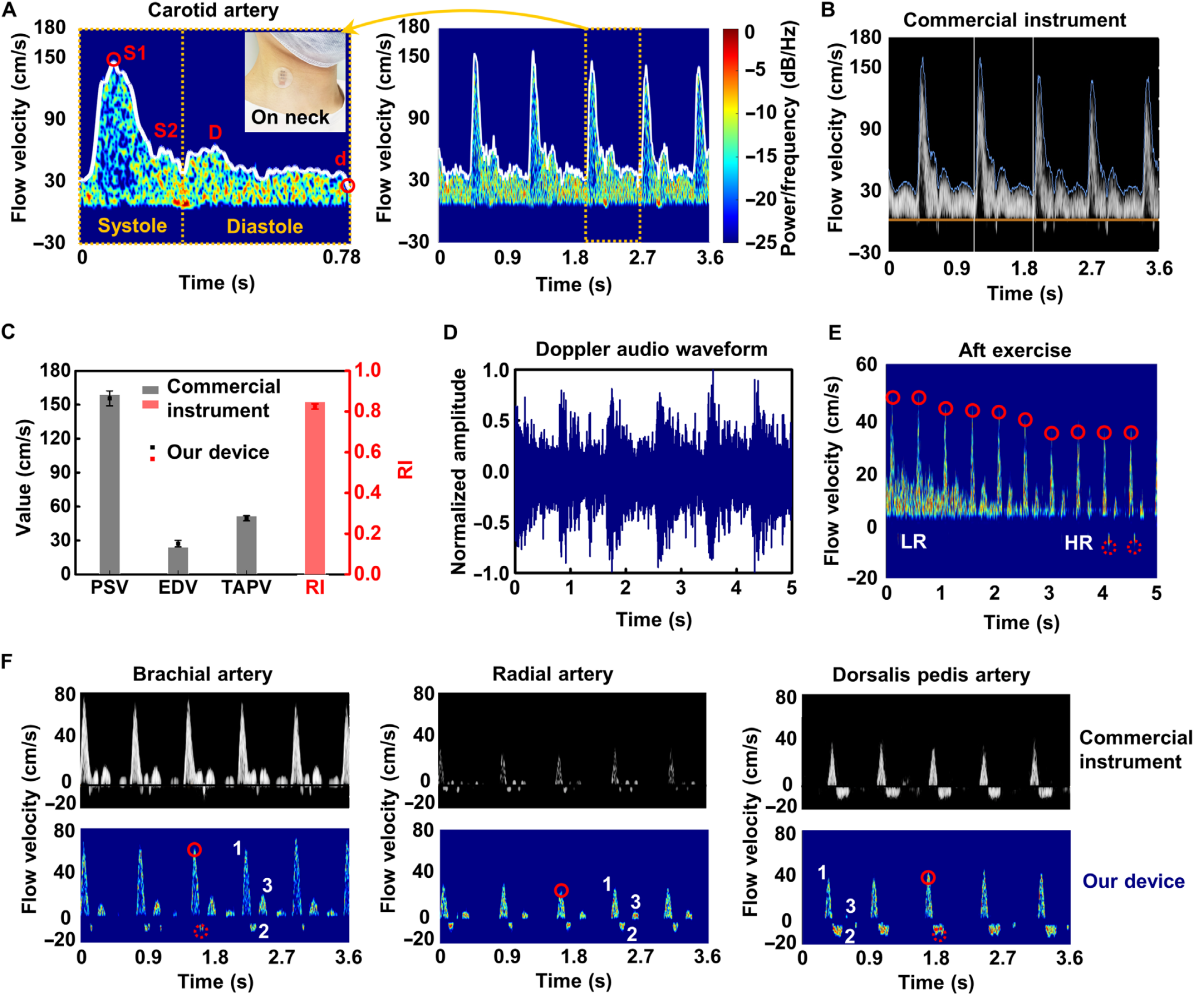
Blood flow velocity monitoring. (**A**) Typical carotid blood flow spectra during a cardiac cycle (left) and several cycles (right). Feature points are marked in the left image. Insets: An image showing the device mounted on the neck. (**B**) Spectrum of carotid blood flow from a commercial ultrasound machine. (**C**) Calculated key blood flow parameters compared with those obtained by the commercial instrument. Error bars represent ±SD (*N* = 5). (**D**) Doppler audio waveform of measured carotid blood flow. (**E**) Continuous measurement of radial artery blood flow after exercise. The flow waveform gradually transitions from a low-pulsatility monophasic pattern [low resistance (LR)] to a high-pulsatility pattern [high resistance (HR)]. All the spectra of (A) to (F) use the same color bar as in (A). (**F**) Measurements of peripheral arteries (bottom row) and validation (top row) by a commercial instrument. Columns (left to right): brachial artery, radial artery, and dorsalis pedis artery. The small red circles represent the PSV (solid line) and maximum reverse flow velocity (dotted line). The numbers 1, 2, and 3 represent the three phases of blood flow.

Compared with constant flow, blood flow is pulsatile, producing a jumble of Doppler frequency shifts that changes from moment to moment. Spectrum analysis provides quantitative information for blood vessels. We first show an accurate measurement of the carotid blood flow velocity and compare it with the result of a commercial ultrasound machine. The carotid artery is approximately 25 mm below the surface of the skin (*23*). It transports the blood pumped from the heart to the brain and therefore is positively associated with cerebral blood flow (*34*). A typical carotid blood flow spectrum was measured using our device, showing five feature points (Fig. 4A). The first peak systolic velocity (PSV) was the peak velocity *S*_1_, which can be used as an ejection parameter in cardiac systole (*35*). The velocity in the late systole was augmented as the second systolic velocity *S*_2_. The peak diastolic velocity, *D*, was the maximum velocity, which increased owing to the elastic recoil of arteries during diastole. *I* was the incisura between systole and diastole, and *d* represented the end-diastolic velocity (EDV) (*36*). We used an EPIQ 7 ultrasound machine (Philips Ultrasound) for validation, which exhibited remarkable correspondence (Fig. 4B). From various combinations of the PSV, EDV, and time-averaged peak velocity (TAPV), some blood velocity indices or ratios can be derived, such as the resistance index (RI), which is widely used to measure the pulsatility, i.e., the resistance to blood flow, and is suggested to be used for analyzing arteries with continuous forward flow throughout diastole, such as the carotid artery (*37*). The RI is defined as

$$\text{RI}=\frac{\text{PSV}-\text{EDV}}{\text{PSV}}$$

and ranges between 0 and 1. The blood flow parameters calculated from spectrum, including PSV, EDV, TAPV, and RI, were compared with those obtained by a commercial ultrasound machine. The results strongly proved the accuracy of blood flow measurement by our device (Fig. 4C). In addition to the spectrum, the measurement results can be displayed in the form of Doppler audio because Doppler shifts are in the audible range (from 20 Hz to 20 kHz) (Fig. 4D and movie S1). Doing so takes advantage of the human ear’s capabilities to aurally identify certain features of the Doppler flow signal. For instance, a distinctive whining or whistling sound is heard in severe carotid artery stenosis (*1*).

The pulsatility of blood flow in arteries varies from center to periphery. The carotid blood flow in Fig. 4A shows a low-pulsatility waveform with broad systolic peaks and persistent forward flow throughout diastole. The term waveform means the shape of the “wave” on the Doppler frequency spectrum. Waveforms obtained from peripheral arteries have a different appearance, with narrow systolic peaks and reversed or absent diastolic flow due to high-resistance distal vascular beds (*13*). A typical example is the triphasic waveform of blood flow in an extremity artery at rest. The first phase (1) is systolic forward flow, the second phase (2) is brief diastolic flow reversal, and the third phase (3) is diastolic forward flow. Sometimes the late diastolic component is absent in a biphasic waveform. We used a commercial ultrasound machine (Fig. 4F, top row) and the flexible device (Fig. 4F, bottom row) to verify the small peripheral arterial blood flow, including the brachial, radial, and dorsalis pedis arteries. High-pulsatility waveforms of peripheral artery flow were observed. Arteries closer to the distal end, such as the radial and dorsalis pedis, have smaller diameters and higher peripheral resistance, exhibiting a decrease in PSV and relatively large reverse blood flow. The results of the commercial ultrasound machine and the flexible device showed good consistency.

The Doppler spectrum contains abundant information related to factors affecting cardiovascular health, such as age, sex, body height, and weight (*38*). Changes in both physiological and pathological conditions will alter peripheral resistance and flow patterns. The triphasic waveform in extremity arteries at rest converts to a low-resistance, monophasic pattern after exercise because the capillary bed opens and peripheral resistance is reduced (*1*). Figure 4E shows the continuous measurement of radial artery blood flow after exercise. The flow waveform gradually transitions from a low-pulsatility monophasic pattern to a high-pulsatility pattern, and last, reverse flow appears. The PSV gradually decreases because cardiac output decreases and flow resistance increases. The device can continuously illustrate changes in vascular resistance, proving its role in capturing the characteristics of blood flow.

### Cuff experiments

**Fig. 5.**
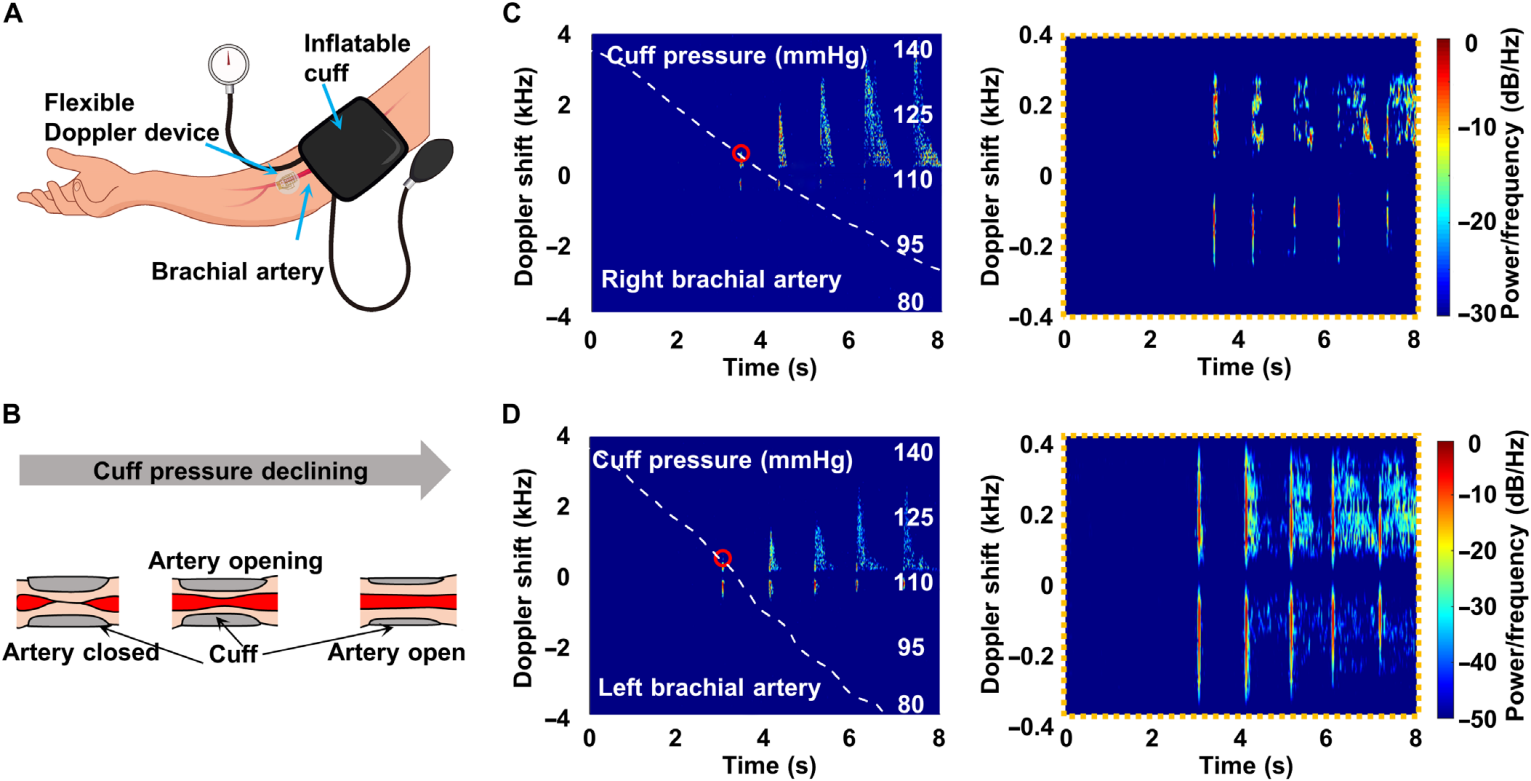
Monitoring of blood flow and artery wall motion in cuff experiments. (**A**) Measurement setup, with our device and an inflatable cuff wrapped around the upper arm. (**B**) Schematic of the measurement. The cuff is initially inflated above 140 mmHg to occlude arterial flow. Then, the artery reopens, and blood flow resumes at some point as the cuff pressure declines. (**C** and **D**) Doppler spectra and cuff pressure variations (white dashed line) of the right brachial artery (C) and left brachial artery (D). The graphs in the right panels of (B) and (C) show the motion of the artery wall. The small red circles represent the systolic arterial pressure.

We used a flexible Doppler device to monitor the behavior of the brachial artery from the fully closed state to an open state. A schematic of the measurement setup is shown in Fig. 5A. An inflatable cuff was wrapped around the upper arm. It was then inflated well above 140 mmHg to occlude arterial flow and then slowly deflated. At some point, the artery reopened, and blood flow resumed (Fig. 5B). When the cuff pressure was high, the vessel was closed, and there was no Doppler shift signal that could be detected. With the decrease in cuff pressure, shift signals appeared suddenly at a certain pressure level, obvious blood flow signals occurred, and the peak shift gradually increased (left side of Fig. 5, C and D). The first marked shift signal in this process, that is, the bright short lines in the low-frequency zone in the figure, was mainly caused by the movement of the artery wall. The cuff pressure at it equaled the systolic blood pressure. The relatively small motion of an uncompressed brachial artery does not generate a detectable Doppler shift signal, but if a cuff wrapped around the arm is inflated to a level between the systolic and diastolic arterial pressures, the artery wall will generate distinctive Doppler shift signals, and it opens much more abruptly than it closes (*39*). On the right side of Fig. 5 (B and C), the spectrum of the motion signal of the artery wall below 400 Hz is shown after filtering out blood flow signals with high frequency shifts. With the opening of the blood vessel, the blood flow gradually resumed, and the peak frequency shift of the vessel wall decreased. The signal as the artery opened was more obvious than the signal of blood flow, but the closure signal is difficult to detect because of its low frequency shift (*39*), which can be solved by increasing the transmit frequency of the piezoelectric transducer or reducing its inclination angle (because the artery walls move perpendicular to the direction of blood flow). Movie S2 shows Doppler audio during this opening of the brachial artery in the right arm.

## DISCUSSION

The flexible and lightweight Doppler ultrasound device supports noninvasive, real-time, and continuous monitoring of blood flow velocity in human arteries. Compared to the clinical ultrasound machine, the device avoids complex imaging (for Doppler angle measuring), requires no experienced operator, applies much smaller pressure, and thus helps with long-term monitoring. Compared to other blood flow monitoring technologies, it can provide the absolute velocities of all moving scatterers in the sample range and require no calibration by using the DBUD method. Strategic designs of the material composition and the microfabrication procedure ensure that it can be attached to the curved skin surface and provide satisfactory signal quality with no coupling agent. The penetration performance of ultrasound makes detecting arterial blood flow velocity possible at a depth of at least 25 mm. The previously unidentified application, angled array design with DBUD method, and practical fabrication process make the device an advance from the prior flexible ultrasound devices (*23*, *40*). The device could be used for blood flow velocity monitoring after vascular reconstruction procedures or preventive home care exams of some chronic vessel diseases, such as diabetic foot.

Motion artifacts in real-world conditions will bring large noise in the velocity measurement at present. Experiments in our work require the subject to keep the measured position still. The problem needs to be studied further in the future. Future work also includes integrating post-end functions, such as analog circuits, signal processing, analog-to-digital converters, wireless communication, and a power supply, to improve the overall wearability and reduce system power consumption. Piezoelectric transducers with a higher working frequency and smaller inclination angles can be used to further reduce the thickness of the device. For example, a 7.5-MHz piezoelectric transducer could be used, with a size of 1.5 mm by 1.5 mm by 0.2 mm, an inclination angle of 10°, and a height of only approximately 460 μm. A higher driving voltage enables a larger detection depth.

## METHODS

### Fabrication of flexible Doppler ultrasonic device

#### Flexible and stretchable conduct wire

The fabrication process started with the preparation of PI with a thickness of 10 μm by spin coating on a silicon wafer. The PI film was cured at 80°C for 10 min, 120°C for 10 min, and 140°C for 30 min. Cu was deposited with a thickness of 400 nm on the wafer by an electron beam. Then, it was etched into designed patterns by photolithography with a photoresist (AZ5214E) as the mask. Thus, we obtained a PI film with a shaped Cu conductor.

#### Fabrication of soft substrates

Two Cu molds for the top and bottom substrates were processed by machining. Ecoflex (00-30, Smooth-On) was then poured into them to undergo 3 hours of curing. The top and bottom substrates were then peeled off from the molds.

#### Integration

The PI film was transferred to the substrates. Then, two films with top and bottom electrodes were patterned by dry etching with reactive ion etching. Nine 1-3 composite piezoelectric transducers (Baoding Xinwei Dianzi Technology Co. Ltd.) and two copper patches were bonded to the bottom electrode on a soft substrate by conductive silver paint (Conduction CD-03). Next, the top electrode was bonded to the transducers and copper patches, and then, they were cured at 70°C for half an hour. Last, the device was encapsulated in a designed mold with Ecoflex and vacuumized to remove any interfacial gaps (fig. S2). Curing was performed at room temperature for 3 hours.

### Measurement and data analysis of the blood flow velocity

The blood flow velocity measurement was carried out on a healthy male aged 26 under the approval of the Institutional Review Board (IRB) of Tsinghua University, Beijing. The device was activated by sinusoidal excitation (waveform generator DG1000; RIGOL Technologies Inc.) at a peak-to-peak voltage of 10 V. The echo signal was received by an oscilloscope (PXI-5922) with a sampling rate of 15 MHz after preamplification (CTS-8682, Guangdong Goworld Co. Ltd.). An ultrasound machine (EPIQ 7, Philips Ultrasound) was used to validate the measurement results. The Doppler spectrum was plotted by using the jet colormap in MATLAB R2020b.

### Contact pressure measurements

The comparison of the contact pressure on skin when using the commercial probe and flexible Doppler device was measured on the forearm of a healthy 26-year-old male. A 5-MHz double-crystal Doppler probe (Lanhui Chaosheng Company) was held by hand to detect the blood flow in the radial artery. In the meantime, a thin-film pressure sensor (RFP602, Yubo Intelligent Technology Co. Ltd.) was bonded in the surface of the probe transducer. We used a DC resistance meter (TH2515, Tonghui Electronic, China) to measure the sensor resistance and transform it to pressure. According to the area of the pressure sensor, the measured pressure value can be transformed to the intensity of pressure. The intensity of pressure of our device on skin was concluded from its weight divided by the area.

### Cuff experiments

The monitoring of blood flow and artery wall motion was assessed on a healthy 24-year-old male lying on a bed under the approval of the IRB of Tsinghua University, Beijing. A sphygmomanometer (Yuwell Medical) with an inflatable cuff and a manometer was used.

## Supplementary Materials

### This PDF file includes:

Notes S1 to S6 Figs. S1 to S6 References

## References

[1] W. J. Zwiebel, J. S. Pellerito, *Introduction to Vascular Ultrasonography* (Saunders, 2005).

[2] D. M. Wootton, D. N. Ku, Fluid mechanics of vascular systems, diseases, and thrombosis. Annu. Rev. Biomed. Eng. 1, 299–329 (1999). 11701491 10.1146/annurev.bioeng.1.1.299

[3] N. R. Neyra, T. A. Ikizler, R. E. May, J. Himmelfarb, G. Schulman, Y. Shyr, R. M. Hakim, Change in access blood flow over time predicts vascular access thrombosis. Kidney Int. 54, 1714–1719 (1998). 9844149 10.1046/j.1523-1755.1998.00145.x

[4] W. M. Blackshear, D. J. Phillips, P. M. Chikos, J. D. Harley, B. L. Thiele, D. E. Strandness Jr., Carotid artery velocity patterns in normal and stenotic vessels. Stroke 11, 67–71 (1980). 7355433 10.1161/01.str.11.1.67

[5] C. K. Zarins, D. P. Giddens, B. K. Bharadvaj, V. S. Sottiurai, R. F. Mabon, S. Glagov, Carotid bifurcation atherosclerosis. Quantitative correlation of plaque localization with flow velocity profiles and wall shear stress. Circ. Res. 53, 502–514 (1983). 6627609 10.1161/01.res.53.4.502

[6] P. A. Schneider, M. E. Rossman, E. F. Bernstein, S. Torem, E. B. Ringelstein, S. M. Otis, Effect of internal carotid artery occlusion on intracranial hemodynamics. Transcranial Doppler evaluation and clinical correlation. Stroke 19, 589–593 (1988). 3284020 10.1161/01.str.19.5.589

[7] R. C. Webb, Y. Ma, S. Krishnan, Y. Li, S. Yoon, X. Guo, X. Feng, Y. Shi, M. Seidel, N. H. Cho, J. Kurniawan, J. Ahad, N. Sheth, J. Kim, J. G. Taylor VI, T. Darlington, K. Chang, W. Huang, J. Ayers, A. Gruebele, R. M. Pielak, M. J. Slepian, Y. Huang, A. M. Gorbach, J. A. Rogers, Epidermal devices for noninvasive, precise, and continuous mapping of macrovascular and microvascular blood flow. Sci. Adv. 1, e1500701 (2015). 26601309 10.1126/sciadv.1500701 PMC4646823

[8] S. D. Shpilfoygel, R. A. Close, D. J. Valentino, G. R. Duckwiler, X-ray videodensitometric methods for blood flow and velocity measurement: A critical review of literature. Med. Phys. 27, 2008–2023 (2000). 11011728 10.1118/1.1288669

[9] A. Nanda, *Complications in Neurosurgery E-Book* (Elsevier Health Sciences, 2018).

[10] W. M. Novick, J. J. Millili, P. Nemir Jr., Management of acute postoperative thrombosis following carotid endarterectomy. Arch. Surg. 120, 922–925 (1985). 4015383 10.1001/archsurg.1985.01390320046009

[11] G. J. de Borst, F. L. Moll, H. D. W. M. van de Pavoordt, H. W. Mauser, J. C. Kelder, R. G. A. Ackerstaf, Stroke from carotid endarterectomy: When and how to reduce perioperative stroke rate? Eur. J. Vasc. Endovasc. Surg. 21, 484–489 (2001). 11397020 10.1053/ejvs.2001.1360

[12] P. P. Goodney, B. W. Nolan, J. Eldrup-Jorgensen, D. S. Likosky, J. L. Cronenwett; Vascular Study Group of Northern New England, Restenosis after carotid endarterectomy in a multicenter regional registry. J. Vasc. Surg. 52, 897–905.e892 (2010). 20620001 10.1016/j.jvs.2010.05.005

[13] A. Thrush, T. Hartshorne, *Vascular Ultrasound E-Book: How, Why and When* (Elsevier Health Sciences, 2009).

[14] D. Froemel, S. J. Fitzsimons, J. Frank, M. Sauerbier, A. Meurer, J. H. Barker, A Review of thrombosis and antithrombotic therapy in microvascular surgery. Eur. Surg. Res. 50, 32–43 (2013). 23548333 10.1159/000347182

[15] K. R. Eberlin, N. Chen, *Revascularization and Replantation in the Hand, An Issue of Hand Clinics, Ebook* (Elsevier Health Sciences, 2019). 10.1016/j.hcl.2019.01.005 30928055

[16] C. M. Boutry, L. Beker, Y. Kaizawa, C. Vassos, H. Tran, A. C. Hinckley, R. Pfattner, S. Niu, J. Li, J. Claverie, Z. Wang, J. Chang, P. M. Fox, Z. Bao, Biodegradable and flexible arterial-pulse sensor for the wireless monitoring of blood flow. Nat. Biomed. Eng. 3, 47–57 (2019). 30932072 10.1038/s41551-018-0336-5

[17] W. M. Swartz, N. F. Jones, L. Cherup, A. Klein, W. W. Shaw, Direct monitoring of microvascular anastomoses with the 20-MHz ultrasonic doppler probe: An experimental and clinical study. Plast. Reconstr. Surg. 81, 149–161 (1988). 3336646 10.1097/00006534-198802000-00001

[18] T. J. Bill, P. A. Foresman, G. T. Rodeheaver, D. B. Drake, Fibrin sealant: A novel method of fixation for an implantable ultrasonic microdoppler probe. J. Reconstr. Microsurg. 17, 257–262 (2001). 11396587 10.1055/s-2001-14517

[19] J. M. Smit, I. S. Whitaker, A. G. Liss, T. Audolfsson, M. Kildal, R. Acosta, Post operative monitoring of microvascular breast reconstructions using the implantable Cook–Swartz doppler system: A study of 145 probes & technical discussion. J. Plast. Reconstr. Aesthet. Surg. 62, 1286–1292 (2009). 18675608 10.1016/j.bjps.2008.06.007

[20] J. Allen, Photoplethysmography and its application in clinical physiological measurement. Physiol. Meas. 28, R1–R39 (2007). 17322588 10.1088/0967-3334/28/3/R01

[21] S. R. Krishnan, T. R. Ray, A. B. Ayer, Y. Ma, P. Gutruf, K. Lee, J. Y. Lee, C. Wei, X. Feng, B. Ng, Z. A. Abecassis, N. Murthy, I. Stankiewicz, J. Freudman, J. Stillman, N. Kim, G. Young, C. Goudeseune, J. Ciraldo, M. Tate, Y. Huang, M. Potts, J. A. Rogers, Epidermal electronics for noninvasive, wireless, quantitative assessment of ventricular shunt function in patients with hydrocephalus. Sci. Transl. Med. 10, eaat8437 (2018). 30381410 10.1126/scitranslmed.aat8437

[22] G. R. Bashford, Ultrasonic measurement of blood flow velocity and applications for cardiovascular assessments, in *Biomarkers in Cardiovascular Disease*, V. B. Patel, V. R. Preedy, Eds. (Springer, 2015), pp. 1–31.

[23] C. Wang, X. Li, H. Hu, L. Zhang, Z. Huang, M. Lin, Z. Zhang, Z. Yin, B. Huang, H. Gong, S. Bhaskaran, Y. Gu, M. Makihata, Y. Guo, Y. Lei, Y. Chen, C. Wang, Y. Li, T. Zhang, Z. Chen, A. P. Pisano, L. Zhang, Q. Zhou, S. Xu, Monitoring of the central blood pressure waveform via a conformal ultrasonic device. Nat. Biomed. Eng. 2, 687–695 (2018). 30906648 10.1038/s41551-018-0287-x PMC6428206

[24] Y. Ma, Y. Zhang, S. Cai, Z. Han, X. Liu, F. Wang, Y. Cao, Z. Wang, H. Li, Y. Chen, X. Feng, Flexible hybrid electronics for digital healthcare. Adv. Mater. 32, 1902062 (2020). 10.1002/adma.201902062 31243834

[25] S. Yin, Y. Su, A traction-free model for the tensile stiffness and bending stiffness of laminated ribbons of flexible electronics. J. Appl. Mech. 86, 051011 (2019).

[26] P. Pan, Z. Bian, X. Song, X. Zhou, Properties of porous PDMS and stretchability of flexible electronics in moist environment. J. Appl. Mech. 87, 101009 (2020).

[27] K. Kwon, H. Wang, J. Lim, K. S. Chun, H. Jang, I. Yoo, D. Wu, A. J. Chen, C. G. Gu, L. Lipschultz, J. U. Kim, J. Kim, H. Jeong, H. Luan, Y. Park, C.-J. Su, Y. Ishida, S. R. Madhvapathy, A. Ikoma, J. W. Kwak, D. S. Yang, A. Banks, S. Xu, Y. Huang, J.-K. Chang, J. A. Rogers, Wireless, soft electronics for rapid, multisensor measurements of hydration levels in healthy and diseased skin. Proc. Natl. Acad. Sci. U.S.A. 118, e2020398118 (2021). 33468630 10.1073/pnas.2020398118 PMC7865173

[28] A. Y. Rwei, W. Lu, C. Wu, K. Human, E. Suen, D. Franklin, M. Fabiani, G. Gratton, Z. Xie, Y. Deng, S. S. Kwak, L. Li, C. Gu, A. Liu, C. M. Rand, T. M. Stewart, Y. Huang, D. E. Weese-Mayer, J. A. Rogers, A wireless, skin-interfaced biosensor for cerebral hemodynamic monitoring in pediatric care. Proc. Natl. Acad. Sci. U.S.A. 117, 31674–31684 (2020). 33257558 10.1073/pnas.2019786117 PMC7749320

[29] S. Kim, B. Lee, J. T. Reeder, S. H. Seo, S.-U. Lee, A. Hourlier-Fargette, J. Shin, Y. Sekine, H. Jeong, Y. S. Oh, A. J. Aranyosi, S. P. Lee, J. B. Model, G. Lee, M.-H. Seo, S. S. Kwak, S. Jo, G. Park, S. Han, I. Park, H.-I. Jung, R. Ghaffari, J. Koo, P. V. Braun, J. A. Rogers, Soft, skin-interfaced microfluidic systems with integrated immunoassays, fluorometric sensors, and impedance measurement capabilities. Proc. Natl. Acad. Sci. U.S.A. 117, 27906–27915 (2020). 33106394 10.1073/pnas.2012700117 PMC7668081

[30] T. L. Szabo, Doppler modes, in *Diagnostic Ultrasound Imaging*, T. L. Szabo, Ed. (Academic Press, 2004), pp. 337–380.

[31] S. Bjaerum, H. Torp, K. Kristoffersen, Clutter filters adapted to tissue motion in ultrasound color flow imaging. IEEE Trans. Ultrason. Ferroelectr. Freq. Control 49, 693–704 (2002). 12075963 10.1109/tuffc.2002.1009328

[32] R. Moraes, N. Aydin, D. Evans, The performance of three maximum frequency envelope detection algorithms for Doppler signals. J. Vasc. Invest. 1, 126–134 (1995).

[33] P. Bass, S. Burroughs, N. Carr, C. Way, *Master Medicine: General and Systematic Pathology E-Book* (Elsevier Health Sciences, 2008).

[34] K. Abe, H. Iwanaga, E. Inada, Effect of nicardipine and diltiazem on internal carotid artery blood flow velocity and local cerebral blood flow during cerebral aneurysm surgery for subarachnoid hemorrhage. J. Clin. Anesth. 6, 99–105 (1994). 8204245 10.1016/0952-8180(94)90004-3

[35] D. Maulik, Spectral Doppler sonography: Waveform analysis and hemodynamic interpretation, in *Doppler Ultrasound in Obstetrics and Gynecology*, D. Maulik, Ed. (Springer, 2005), pp. 35–56.

[36] A. Azhim, M. Katai, M. Akutagawa, Y. Hirao, K. Yoshizaki, S. Obara, M. Nomura, H. Tanaka, H. Yamaguchi, Y. Kinouchi, Exercise improved age-associated changes in the carotid blood velocity waveforms. J. Biomed. Pharm. Eng. 1, 17–26 (2007).

[37] C. Oates, *Cardiovascular Haemodynamics and Doppler Waveforms Explained* (Cambridge Univ. Press, 2001).

[38] A. R. Rasyada, A. Azran, Flow velocity in common carotid artery, in *Carotid Artery—Gender and Health* (IntechOpen, 2019).

[39] H. F. Stegall, M. B. Kardon, W. T. Kemmerer, Indirect measurment of arterial blood pressure by Doppler ultrasonic sphygmomanometry. J. Appl. Physiol. 25, 793–798 (1968). 5727212 10.1152/jappl.1968.25.6.793

[40] H. Hu, X. Zhu, C. Wang, L. Zhang, X. Li, S. Lee, Z. Huang, R. Chen, Z. Chen, C. Wang, Y. Gu, Y. Chen, Y. Lei, T. Zhang, N. Kim, Y. Guo, Y. Teng, W. Zhou, Y. Li, A. Nomoto, S. Sternini, Q. Zhou, M. Pharr, F. L. di Scalea, S. Xu, Stretchable ultrasonic transducer arrays for three-dimensional imaging on complex surfaces. Sci. Adv. 4, eaar3979 (2018). 29740603 10.1126/sciadv.aar3979 PMC5938227

[41] P. N. T. Wells, Physical principles of ultrasonic diagnosis. Med. Biol. Eng. 8, 219 (1970).

[42] H. Yuk, S. Lin, C. Ma, M. Takaffoli, N. X. Fang, X. Zhao, Hydraulic hydrogel actuators and robots optically and sonically camouflaged in water. Nat. Commun. 8, 14230 (2017). 28145412 10.1038/ncomms14230 PMC5296644

[43] T. Chiu, Z. Xiong, D. Parsons, M. R. Folkert, P. M. Medin, B. Hrycushko, Low-cost 3D print–based phantom fabrication to facilitate interstitial prostate brachytherapy training program. Brachytherapy 19, 800–811 (2020). 32690386 10.1016/j.brachy.2020.06.015

[44] N. Cardim, H. Dalen, J.-U. Voigt, A. Ionescu, S. Price, A. N. Neskovic, T. Edvardsen, M. Galderisi, R. Sicari, E. Donal, A. Stefanidis, V. Delgado, J. Zamorano, B. A. Popescu, The use of handheld ultrasound devices: A position statement of the European Association of Cardiovascular Imaging (2018 update). Eur. Heart J. Cardiovasc. Imaging 20, 245–252 (2018). 10.1093/ehjci/jey145 30351358

