# Supplementary material for: Flexible Doppler ultrasound device for the monitoring of blood flow velocity
Source: Sci Adv. 2021 Oct 27;7(44):eabi9283. doi: 10.1126/sciadv.abi9283 (PMC8550238; doi:10.1126/sciadv.abi9283)
Supplement: Supplementary file 1 — Notes S1 to S6 Figs. S1 to S6 References [file sciadv.abi9283_sm.pdf]

Science Advances
AAAS

Supplementary Materials for

Flexible Doppler ultrasound device for the monitoring of blood flow velocity

Fengle Wang, Peng Jin, Yunlu Feng, Ji Fu, Peng Wang, Xin Liu, Yingchao Zhang, Yinji Ma, Yingyun Yang, Aiming Yang, Xue Feng*

*Corresponding author. Email: fengxue@tsinghua.edu.cn

Published 27 October 2021, *Sci. Adv.* **7**, eabi9283 (2021)
DOI: 10.1126/sciadv.abi9283

The PDF file includes:

Notes S1 to S6
Figs. S1 to S6
References

Other Supplementary Material for this manuscript includes the following:

Movies S1 and S2

Supplementary note 1: Signal processing procedure

The signal processing procedure is shown in Supplementary Fig. 3. We use quadrature detection to obtain the Doppler signal of moving scatterers (*1, 30*). The received digital signal is first processed through a bandpass filter between 3 MHz and 6 MHz. Then, it is mixed with the emission signal to produce two identical outputs (IQ signals) shifted 90 degrees relative to each other. The IQ signals can still be quite ripply at twice the carrier frequency, requiring a reasonable amount of 500 kHz low-pass filtering before the spectrum is constructed. Then, they are downsampled to a sampling rate of 12000 Hz. A cluttered signal from slowly moving muscular tissue, including vessel walls, which is stronger, with a lower frequency than that of the signal from blood, remains (*31*). Therefore, a high-pass filter above 150 Hz is used to separate the signals from blood and tissue. The obtained IQ signals are combined as $I + iQ$ (i is the imaginary unit). Next, the complex signal is analyzed by the power spectral density (PSD), or the Doppler audio can be derived from the Hilbert transform (*30*). Finally, we used common logarithmic compression to process the PSD result to reduce the dynamic range of PSD (*1*), that is

$$P_{\log} = 10 \log_{10} \frac{P}{P_{\max}}$$

where P_{max} is the maximum PSD value.

Supplementary note 2: Choice of device design and emission mode

Two ways of Doppler modes are generally used. A pulsed wave (PW) Doppler system is excited by repetitive short-duration bursts rather than continuous sinusoidal waves, as in the CW system. One transducer transmits ultrasound waves and detects the echo at a certain range gate (*1, 30*). Compared with CW Doppler, PW Doppler allows control of the region of active insonification and calculates only the Doppler spectrum in that region. However, two important reasons exist for why CW Doppler is more preferable than PW Doppler in wearable Doppler devices. First, the gate position and duration need to be adjusted in the PW Doppler system according to the position of the target vessel. Most commercial ultrasound machines use two modes, i.e., B-mode and Doppler by phased array technology, at the same time, which can superimpose a line with a marker indicating the pulse length, range depth, and direction onto a B-mode ultrasound image. Although a flexible ultrasound imaging array has been proposed (*23*), simultaneous B-mode imaging and pulsed Doppler are not practical for wearable flexible ultrasound devices, requiring too many computing resources and a high power consumption and increase the user’s operation difficulty (the gate position may change because of the curved skin surface and individual body structure). Phased array technology also requires accurate information on each transducer’ position, while the flexibility changes the relative positions when the device is attached to the curved skin surface. The CW Doppler system is easier to operate as a result.

Second, CW Doppler has no limited maximum measurable Doppler frequency, while that of PW Doppler must be lower than half the pulse repetition frequency, or it will cause aliasing (*1*). If the pulse repetition frequency is increased, the detectable depth will be reduced. So PW Doppler may not detect peak flow velocity in some arteries, such as carotid artery. Although the CW Doppler method cannot select the sample volume of blood flow for measurement, it can measure various arteries by controlling the emission intensity and calculating the spectral envelope (peak flow velocity) for analysis.

Supplementary note 3: Derivations of DBUD method

According to the Doppler effect and geometrical conditions, we obtained the following equations:

$$\begin{cases} f_1 = \dfrac{2v\sin\gamma_1}{c} f_0 \\ f_2 = \dfrac{2v\sin\gamma_2}{c} f_0 \\ \Theta_1 - \Theta_2 = \gamma_1 - \gamma_2 \end{cases} \tag{1}$$

where

$$\begin{cases} \Theta_1 = \sin^{-1}\left(\dfrac{\sin\theta_1}{\eta}\right) \\ \Theta_2 = \sin^{-1}\left(\dfrac{\sin\theta_2}{\eta}\right) \end{cases} \tag{2}$$

and c is the sound velocity in blood, f_0 is the transmitting frequency, f_1 and f_2 are Doppler frequency shifts generated by two ultrasound beams, and γ_1 and γ_2 are the Doppler angels. θ_1, θ_2 are the inclination angles of the transducers. η is the refractive index which equals the ratio of sound velocities in Ecoflex and skin.

We define

$$\gamma_0 = \frac{\gamma_1 + \gamma_2}{2} \tag{3}$$

$$\Delta\gamma = \frac{\gamma_1 - \gamma_2}{2} = \frac{\Theta_1 - \Theta_2}{2} \tag{4}$$

that is,

$$\begin{cases} \gamma_1 = \gamma_0 + \Delta\gamma \\ \gamma_2 = \gamma_0 - \Delta\gamma \end{cases} \quad (5)$$

Then, we combine equation (5) with equation (1):

$$\begin{cases} \dfrac{cf_1}{2vf_0} = \sin\gamma_1 = \sin(\gamma_0 + \Delta\gamma) = \sin\gamma_0 \cos\Delta\gamma + \cos\gamma_0 \sin\Delta\gamma \\ \dfrac{cf_2}{2vf_0} = \sin\gamma_2 = \sin(\gamma_0 - \Delta\gamma) = \sin\gamma_0 \cos\Delta\gamma - \cos\gamma_0 \sin\Delta\gamma \end{cases} \quad (6)$$

From equation (6), we obtain

$$\begin{cases} \sin\gamma_0 = \dfrac{c}{4vf_0 \cos\Delta\gamma}(f_1 + f_2) \\ \cos\gamma_0 = \dfrac{c}{4vf_0 \sin\Delta\gamma}(f_1 - f_2) \end{cases} \quad (7)$$

Then, we calculate the quadratic sum of the two equations above:

$$\left(\frac{c}{4vf_0 \cos\Delta\gamma}(f_1 + f_2)\right)^2 + \left(\frac{c}{4vf_0 \sin\Delta\gamma}(f_1 - f_2)\right)^2 = 1 \quad (8)$$

We now divide the two equations in (7):

$$\gamma_0 = \arctan\left(\frac{f_1 + f_2}{f_1 - f_2} \tan\Delta\gamma\right) \quad (9)$$

From equation (8), the absolute velocity is obtained:

$$v = \frac{c}{4f_0}\sqrt{\left(\frac{f_1 + f_2}{\cos\Delta\gamma}\right)^2 + \left(\frac{f_1 - f_2}{\sin\Delta\gamma}\right)^2} \quad (10)$$

Substituting equation (9) into (5), Doppler angles γ_1, γ_2 are obtained.

$$\begin{cases} \gamma_1 = \arctan\left(\dfrac{f_1 + f_2}{f_1 - f_2} \tan\Delta\gamma\right) + \Delta\gamma \\ \gamma_2 = \arctan\left(\dfrac{f_1 + f_2}{f_1 - f_2} \tan\Delta\gamma\right) - \Delta\gamma \end{cases} \quad (11)$$

where

$$\Delta\gamma = \frac{\Theta_1 - \Theta_2}{2}$$

According to the geometrical conditions, the inclination angle of the vessel is:

$$\alpha = \Theta_1 - \gamma_1 \tag{12}$$

Supplementary note 4: Choice of working frequency of transducers.

The choice of the working frequency of transducers is governed by competing physical interactions. The intensity of ultrasonic waves scattered from small scatterers such as red blood cells increases rapidly with increasing frequency, being proportional to the frequency raised to the fourth power (*2*). However, the rate of beam attenuation also increases as the frequency increases. Doppler applications in the carotid artery usually employ frequencies of 4 to 5 MHz to avoid significant attenuation losses (*1*). Wells gives a frequency which has optimal signal-to-ratio as 13/R MHz (*41*), where R (cm) is the depth. If R equals to 2.5 cm, the frequency should be 5.2 MHz. Therefore, we chose piezoelectric transducers with a resonant frequency of 5 MHz which can detect the depth of the carotid artery.

Supplementary note 5: Measurement uncertainty.

The measurement uncertainty of peak flow velocity (PFV) was evaluated as follows. The PFV is:

$$V = \frac{cf}{2f_0 \sin\gamma}$$

where c is the sound speed in tissue mimicking material, f is the peak Doppler shift, f_0 is the transmitted frequency, and γ, known as the Doppler angle, is the angle between the axis of the ultrasound beam and the direction of flow, looking toward the transducer. γ is calculated from the inclination angle θ of transducers, sound speed c and c_E for Ecoflex according to the refraction formula:

$$\gamma = \arcsin\left(\frac{c}{c_E}\sin\theta\right)$$

Overall, the relation is shown as follows:

$$V = \frac{cf}{2f_0\left(\frac{c}{c_E}\sin\theta\right)}$$

To evaluate the measurement uncertainty, individual uncertainty of each input variables should be evaluated. We used the standard ultrasound phantom (Optimizer 1425A, Gammex, Inc) with a peak flow velocity (PFV) preset at 100 cm/s.

f is the peak Doppler shift. The A type uncertainty of f is the standard deviation derived from repeated measurements within 3 minutes. The results for 10 times were 2479.8 Hz, 2415.1 Hz, 2493.8 Hz, 2574.3 Hz, 2492.8 Hz, 2578.0 Hz, 2489.1 Hz, 2533.6 Hz, 2503.9 Hz, 2548.4 Hz. The A type uncertainty is

$$U_f = \sqrt{\frac{\sum_{i=1}^{n}(f_i - \bar{f})^2}{n(n-1)}}$$

Therefore, we obtain $U_f = 15.54$ Hz. The relative uncertainty $U_{rel\_f} = \frac{U_f}{\bar{f}} = 0.62\%$.

The sound speed of the tissue mimicking material of the phantom is provided as 1540±10 m/s. It is assumed in uniform distribution. The uncertainty is $U_c = \frac{10}{\sqrt{3}} = 5.7735$ m/s. The relative uncertainty $U_{rel\_c} = \frac{U_c}{\bar{c}} = 0.37\,\%$. The sound speed of Ecoflex silicone is about 1000 cm/s and the maximum error is estimated at less than 20 cm/s (*42, 43*). The uncertainty is $U_{cE} = \frac{20}{\sqrt{3}}/\overline{c_E} = 1.15\%$.

The flexible device avoids the handheld instability of motion, so the fluctuation of inclination angle θ is negligible. We measured the inclination angle of the transducer by using an angle ruler (187-151, San Liang). The results 17.25 °, 17.2 °, 17.2 °, 17.2 °, 17.25 ° have an A type uncertainty $U_{\theta A} = 0.0115°$. The maximum error is 0.05 °. So the B type uncertainty is $U_{\theta B} = \frac{0.05}{\sqrt{3}} = 0.0289$ °. The relative uncertainty $U_{rel\_\theta} = \frac{\sqrt{U_{\theta A}^2 + U_{\theta B}^2}}{\bar{\theta}} = 0.18\%$. The transmitted frequency f_0 is generated by waveform generator which has a high accuracy so its uncertainty is negligible.

The combined measurement uncertainty of V is calculated based on the assumption that there are no correlations between each one of the input variables, since all of them are measured independently. The measurement uncertainty of V can be calculated as:

$$V\left(f, c, c_E, \theta\right) = \frac{cf}{2 f_0 \left(\frac{c}{c_E} \sin\theta\right)}$$

$$U_V = \sqrt{\left(\frac{\partial V}{\partial f} U_f\right)^2 + \left(\frac{\partial V}{\partial c} U_c\right)^2 + \left(\frac{\partial V}{\partial c_E} U_{c_E}\right)^2 + \left(\frac{\partial V}{\partial \theta} U_\theta\right)^2}$$

We used MATLAB R2020b to calculate the combined uncertainty U_V = 1.27 cm/s when the PFV is 100 cm/s. The relative uncertainty is $U_{rel\_V} = 1.27/100 = 1.27\%$. The uncertainty curve in Supplementary Fig. 6 shows the uncertainty as a function of PFV.

Supplementary note 6: Comparison to the handheld ultrasound device.

Recent technological advances in progressive miniaturization of ultrasound machines have led to the development of handheld ultrasound devices (HUD) (*44*). The work principle of them is the same with traditional commercial equipment. They are simplified and miniaturized version. However, there are three significant disadvantages of them.

First, their probes are still rigid and big (generally over 3 cm in width and 10 cm in length) compared with our results, which means they require high handheld stability and can not be worn for long-term recording. With only 750 mg in weight, 1 mm in thickness, and similar elastic modulus to the skin, our flexible device keeps conformal light contact with the curved skin surface and do the measurement without any manual holding.

Second, excess holding forces on the probe will cause damage to the subject during long-term recording (*23*), especially for postoperative patients with fragile skin. The traditional handheld probe can exert over 1000 Pa to the human skin (Fig. 2f). The flexible device induces negligible pressure (15 Pa) to the skin surface.

Third, lots of unpleasantly cold gel that can quickly dry out would be needed during the using of handheld probes, thus limiting their wearability. For our work, silicone substrate is coated at the bottom of the device to remove the influence of the air gaps

thoroughly. Therefore, our flexible device can do the measurement with comparable SNR to the acoustic coupling condition provided by the ultrasound gel (Supplementary Fig. 4).

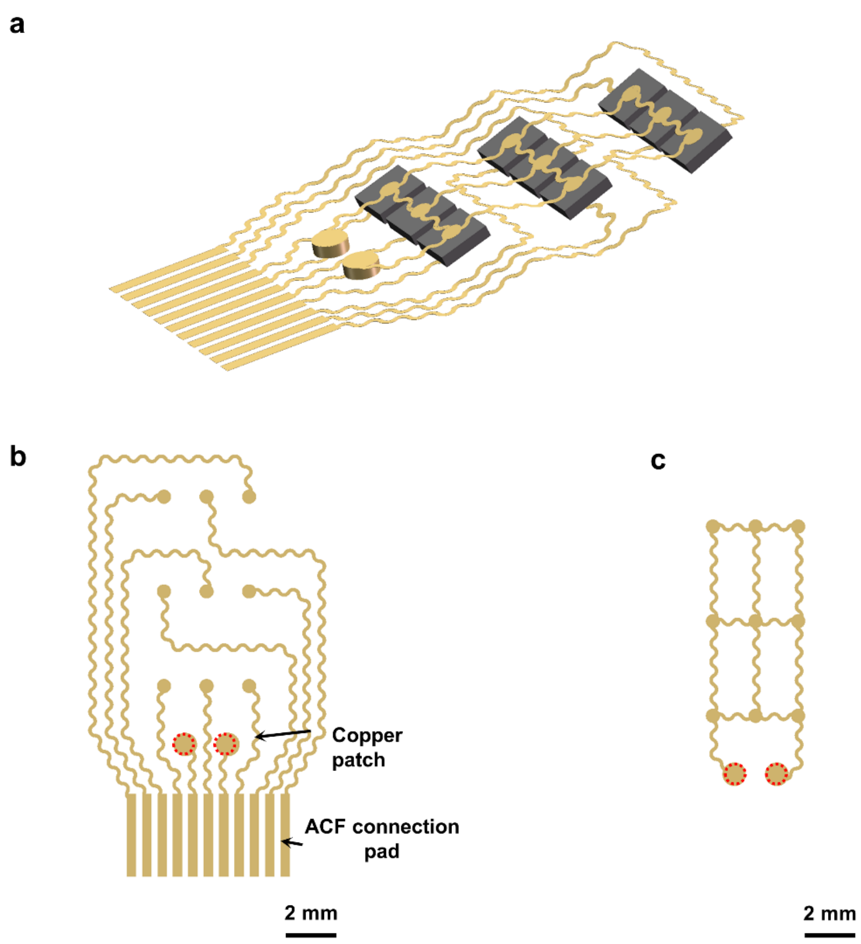

Supplementary Fig 1. Design layout. a, View without silicone package**. b**, Bottom electrode. **c**, Top electrode.

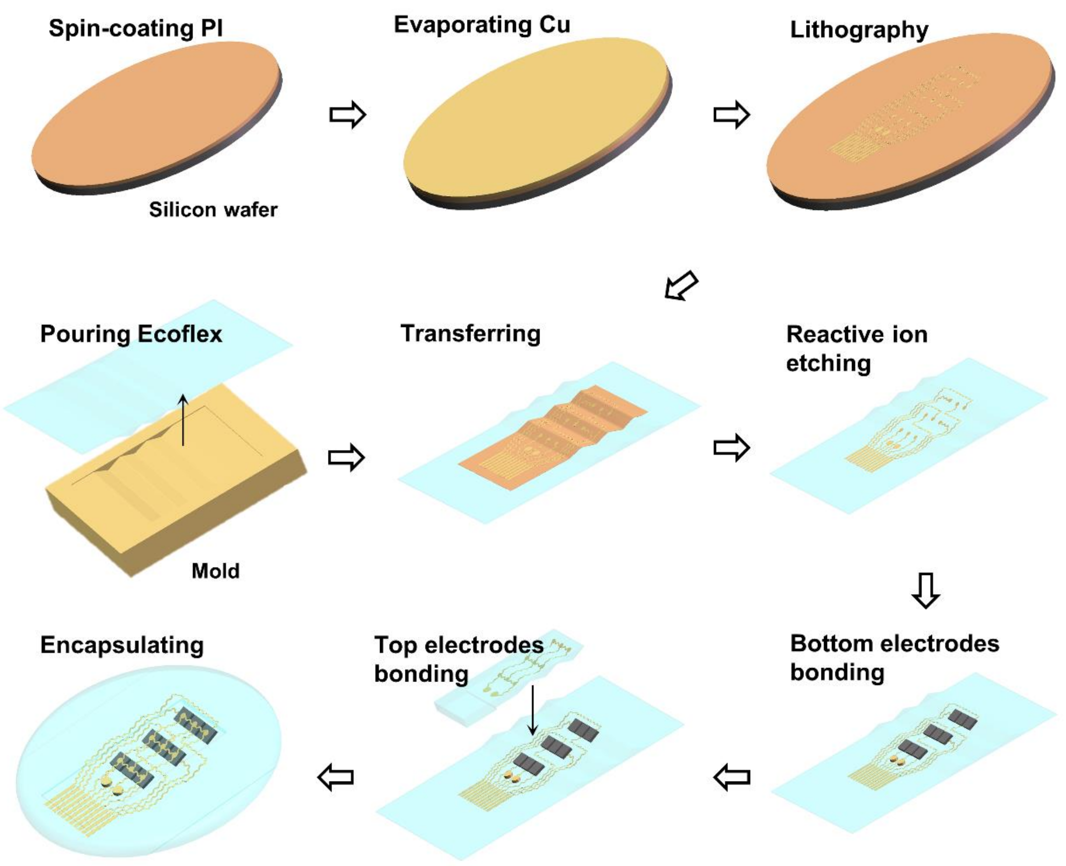

Supplementary Fig. 2. Doppler ultrasound device fabrication flow chart.

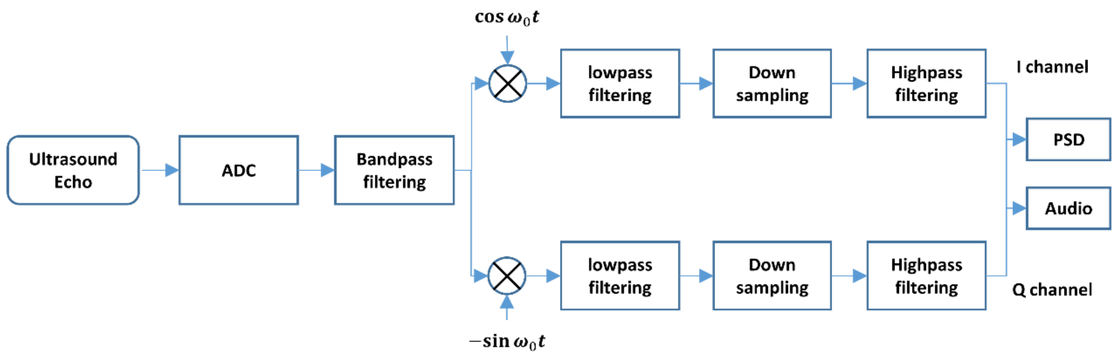

Supplementary Fig. 3. Signal processing procedure. ω_0 is the circular frequency of the emission signal.

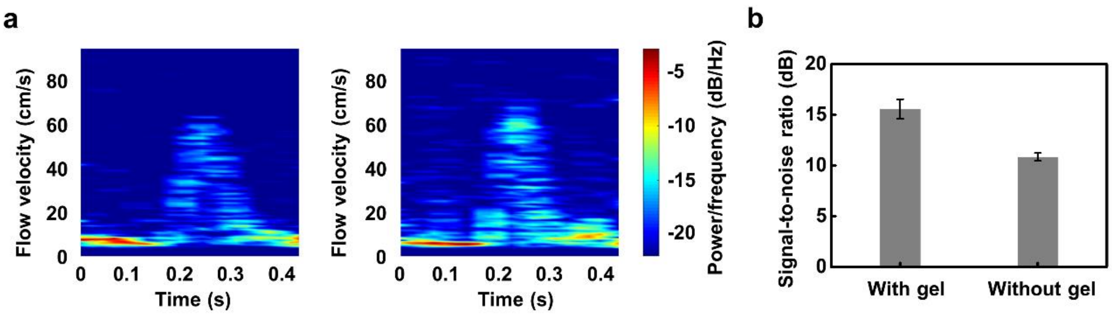

Supplementary Fig. 4. Signal qualities of the conformal ultrasound device with and without coupling agents. a, Spectrum tested from branchial arteries with gel (left) and without gel (right). **b,** Signal quality comparison. The results show that the absence of gel hardly degrades the signal quality.

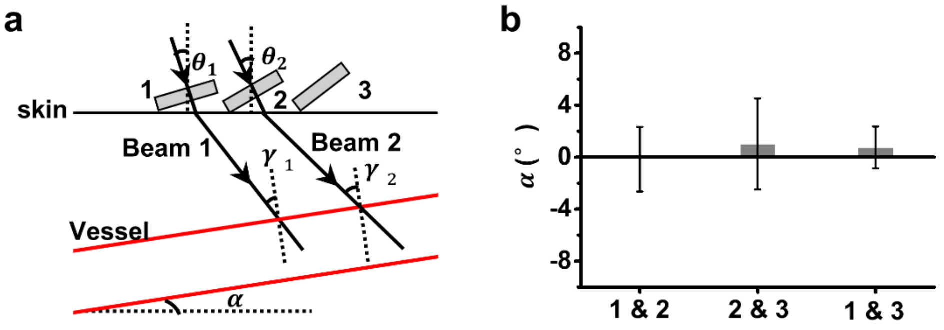

Supplementary Fig. 5. The direction of vessels calculated by using the DBUD method. a, Schematic of the DBUD method. **b,** Calculated results of five experiments. 1&2 represent using transducer 1 and 2. The results show that the inclination angle of vessel α is almost horizontal, which is similar to the reality.

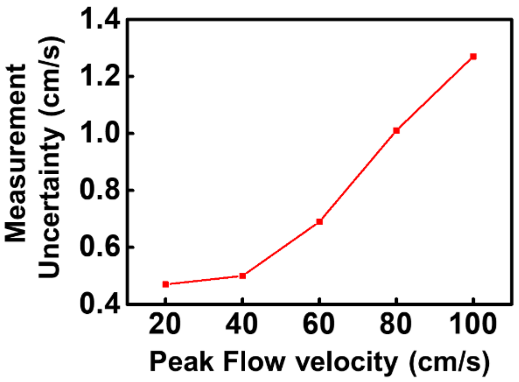

Supplementary Fig. 6. The measurement uncertainty. The results show the uncertainty when measuring the peak flow velocity of ultrasound phantom.

REFERENCES AND NOTES

1. W. J. Zwiebel, J. S. Pellerito, *Introduction to Vascular Ultrasonography* (Saunders, 2005).
2. D. M. Wootton, D. N. Ku, Fluid mechanics of vascular systems, diseases, and thrombosis. *Annu. Rev. Biomed. Eng.* **1**, 299–329 (1999).
3. N. R. Neyra, T. A. Ikizler, R. E. May, J. Himmelfarb, G. Schulman, Y. Shyr, R. M. Hakim, Change in access blood flow over time predicts vascular access thrombosis. *Kidney Int.* **54**, 1714–1719 (1998).
4. W. M. Blackshear, D. J. Phillips, P. M. Chikos, J. D. Harley, B. L. Thiele, D. E. Strandness Jr, Carotid artery velocity patterns in normal and stenotic vessels. *Stroke* **11**, 67–71 (1980).
5. C. K. Zarins, D. P. Giddens, B. K. Bharadvaj, V. S. Sottiurai, R. F. Mabon, S. Glagov, Carotid bifurcation atherosclerosis. Quantitative correlation of plaque localization with flow velocity profiles and wall shear stress. *Circ. Res.* **53**, 502–514 (1983).
6. P. A. Schneider, M. E. Rossman, E. F. Bernstein, S. Torem, E. B. Ringelstein, S. M. Otis, Effect of internal carotid artery occlusion on intracranial hemodynamics. Transcranial Doppler evaluation and clinical correlation. *Stroke* **19**, 589–593 (1988).
7. R. C. Webb, Y. Ma, S. Krishnan, Y. Li, S. Yoon, X. Guo, X. Feng, Y. Shi, M. Seidel, N. H. Cho, J. Kurniawan, J. Ahad, N. Sheth, J. Kim, J. G. Taylor VI, T. Darlington, K. Chang, W. Huang, J. Ayers, A. Gruebele, R. M. Pielak, M. J. Slepian, Y. Huang, A. M. Gorbach, J. A. Rogers, Epidermal devices for noninvasive, precise, and continuous mapping of macrovascular and microvascular blood flow. *Sci. Adv.* **1**, e1500701 (2015).
8. S. D. Shpilfoygel, R. A. Close, D. J. Valentino, G. R. Duckwiler, X-ray videodensitometric methods for blood flow and velocity measurement: A critical review of literature. *Med. Phys.* **27**, 2008–2023 (2000).
9. A. Nanda, *Complications in Neurosurgery E-Book* (Elsevier Health Sciences, 2018).
10. W. M. Novick, J. J. Millili, P. Nemir Jr, Management of acute postoperative thrombosis following carotid endarterectomy. *Arch. Surg.* **120**, 922–925 (1985).
11. G. J. de Borst, F. L. Moll, H. D. W. M. van de Pavoordt, H. W. Mauser, J. C. Kelder, R. G. A. Ackerstaf, Stroke from carotid endarterectomy: When and how to reduce perioperative stroke rate? *Eur. J. Vasc. Endovasc. Surg.* **21**, 484–489 (2001).
12. P. P. Goodney, B. W. Nolan, J. Eldrup-Jorgensen, D. S. Likosky, J. L. Cronenwett; Vascular Study Group of Northern New England, Restenosis after carotid endarterectomy in a multicenter regional registry. *J. Vasc. Surg.* **52**, 897–905.e892 (2010).
13. A. Thrush, T. Hartshorne, *Vascular Ultrasound E-Book: How, Why and When* (Elsevier Health Sciences, 2009).
14. D. Froemel, S. J. Fitzsimons, J. Frank, M. Sauerbier, A. Meurer, J. H. Barker, A Review of thrombosis and antithrombotic therapy in microvascular surgery. *Eur. Surg. Res.* **50**, 32–43 (2013).
15. K. R. Eberlin, N. Chen, *Revascularization and Replantation in the Hand, An Issue of Hand Clinics, Ebook* (Elsevier Health Sciences, 2019).
16. C. M. Boutry, L. Beker, Y. Kaizawa, C. Vassos, H. Tran, A. C. Hinckley, R. Pfattner, S. Niu, J. Li, J. Claverie, Z. Wang, J. Chang, P. M. Fox, Z. Bao, Biodegradable and flexible arterial-pulse sensor for the wireless monitoring of blood flow. *Nat. Biomed. Eng.* **3**, 47–57 (2019).

17. W. M. Swartz, N. F. Jones, L. Cherup, A. Klein, W. W. Shaw, Direct monitoring of microvascular anastomoses with the 20-MHz ultrasonic doppler probe: An experimental and clinical study. *Plast. Reconstr. Surg.* **81**, 149–161 (1988).
18. T. J. Bill, P. A. Foresman, G. T. Rodeheaver, D. B. Drake, Fibrin sealant: A novel method of fixation for an implantable ultrasonic microdoppler probe. *J. Reconstr. Microsurg.* **17**, 257–262 (2001).
19. J. M. Smit, I. S. Whitaker, A. G. Liss, T. Audolfsson, M. Kildal, R. Acosta, Post operative monitoring of microvascular breast reconstructions using the implantable Cook–Swartz doppler system: A study of 145 probes & technical discussion. *J. Plast. Reconstr. Aesthet. Surg.* **62**, 1286–1292 (2009).
20. J. Allen, Photoplethysmography and its application in clinical physiological measurement. *Physiol. Meas.* **28**, R1–R39 (2007).
21. S. R. Krishnan, T. R. Ray, A. B. Ayer, Y. Ma, P. Gutruf, K. Lee, J. Y. Lee, C. Wei, X. Feng, B. Ng, Z. A. Abecassis, N. Murthy, I. Stankiewicz, J. Freudman, J. Stillman, N. Kim, G. Young, C. Goudeseune, J. Ciraldo, M. Tate, Y. Huang, M. Potts, J. A. Rogers, Epidermal electronics for noninvasive, wireless, quantitative assessment of ventricular shunt function in patients with hydrocephalus. *Sci. Transl. Med.* **10**, eaat8437 (2018).
22. G. R. Bashford, Ultrasonic measurement of blood flow velocity and applications for cardiovascular assessments, in *Biomarkers in Cardiovascular Disease*, V. B. Patel, V. R. Preedy, Eds. (Springer, 2015), pp. 1–31.
23. C. Wang, X. Li, H. Hu, L. Zhang, Z. Huang, M. Lin, Z. Zhang, Z. Yin, B. Huang, H. Gong, S. Bhaskaran, Y. Gu, M. Makihata, Y. Guo, Y. Lei, Y. Chen, C. Wang, Y. Li, T. Zhang, Z. Chen, A. P. Pisano, L. Zhang, Q. Zhou, S. Xu, Monitoring of the central blood pressure waveform via a conformal ultrasonic device. *Nat. Biomed. Eng.* **2**, 687–695 (2018).
24. Y. Ma, Y. Zhang, S. Cai, Z. Han, X. Liu, F. Wang, Y. Cao, Z. Wang, H. Li, Y. Chen, X. Feng, Flexible hybrid electronics for digital healthcare. *Adv. Mater.* **32**, 1902062 (2020).
25. S. Yin, Y. Su, A traction-free model for the tensile stiffness and bending stiffness of laminated ribbons of flexible electronics. *J. Appl. Mech.* **86**, 051011 (2019).
26. P. Pan, Z. Bian, X. Song, X. Zhou, Properties of porous PDMS and stretchability of flexible electronics in moist environment. *J. Appl. Mech.* **87**, 101009 (2020).
27. K. Kwon, H. Wang, J. Lim, K. S. Chun, H. Jang, I. Yoo, D. Wu, A. J. Chen, C. G. Gu, L. Lipschultz, J. U. Kim, J. Kim, H. Jeong, H. Luan, Y. Park, C.-J. Su, Y. Ishida, S. R. Madhvapathy, A. Ikoma, J. W. Kwak, D. S. Yang, A. Banks, S. Xu, Y. Huang, J.-K. Chang, J. A. Rogers, Wireless, soft electronics for rapid, multisensor measurements of hydration levels in healthy and diseased skin. *Proc. Natl. Acad. Sci. U.S.A.* **118**, e2020398118 (2021).
28. A. Y. Rwei, W. Lu, C. Wu, K. Human, E. Suen, D. Franklin, M. Fabiani, G. Gratton, Z. Xie, Y. Deng, S. S. Kwak, L. Li, C. Gu, A. Liu, C. M. Rand, T. M. Stewart, Y. Huang, D. E. Weese-Mayer, J. A. Rogers, A wireless, skin-interfaced biosensor for cerebral hemodynamic monitoring in pediatric care. *Proc. Natl. Acad. Sci. U.S.A.* **117**, 31674–31684 (2020).
29. S. Kim, B. Lee, J. T. Reeder, S. H. Seo, S.-U. Lee, A. Hourlier-Fargette, J. Shin, Y. Sekine, H. Jeong, Y. S. Oh, A. J. Aranyosi, S. P. Lee, J. B. Model, G. Lee, M.-H. Seo, S. S. Kwak, S. Jo, G. Park, S. Han, I. Park, H.-I. Jung, R. Ghaffari, J. Koo, P. V. Braun, J. A. Rogers, Soft, skin-interfaced microfluidic systems with integrated immunoassays, fluorometric sensors, and impedance measurement capabilities. *Proc. Natl. Acad. Sci. U.S.A.* **117**, 27906–27915 (2020).

30. T. L. Szabo, Doppler modes, in *Diagnostic Ultrasound Imaging*, T. L. Szabo, Ed. (Academic Press, 2004), pp. 337–380.
31. S. Bjaerum, H. Torp, K. Kristoffersen, Clutter filters adapted to tissue motion in ultrasound color flow imaging. *IEEE Trans. Ultrason. Ferroelectr. Freq. Control* **49**, 693–704 (2002).
32. R. Moraes, N. Aydin, D. Evans, The performance of three maximum frequency envelope detection algorithms for Doppler signals. *J. Vasc. Invest.* **1**, 126–134 (1995).
33. P. Bass, S. Burroughs, N. Carr, C. Way, *Master Medicine: General and Systematic Pathology E-Book* (Elsevier Health Sciences, 2008).
34. K. Abe, H. Iwanaga, E. Inada, Effect of nicardipine and diltiazem on internal carotid artery blood flow velocity and local cerebral blood flow during cerebral aneurysm surgery for subarachnoid hemorrhage. *J. Clin. Anesth.* **6**, 99–105 (1994).
35. D. Maulik, Spectral Doppler sonography: Waveform analysis and hemodynamic interpretation, in *Doppler Ultrasound in Obstetrics and Gynecology*, D. Maulik, Ed. (Springer, 2005), pp. 35–56.
36. A. Azhim, M. Katai, M. Akutagawa, Y. Hirao, K. Yoshizaki, S. Obara, M. Nomura, H. Tanaka, H. Yamaguchi, Y. Kinouchi, Exercise improved age-associated changes in the carotid blood velocity waveforms. *J. Biomed. Pharm. Eng.* **1**, 17–26 (2007).
37. C. Oates, *Cardiovascular Haemodynamics and Doppler Waveforms Explained* (Cambridge Univ. Press, 2001).
38. A. R. Rasyada, A. Azran, Flow velocity in common carotid artery, in *Carotid Artery—Gender and Health* (IntechOpen, 2019).
39. H. F. Stegall, M. B. Kardon, W. T. Kemmerer, Indirect measurment of arterial blood pressure by Doppler ultrasonic sphygmomanometry. *J. Appl. Physiol.* **25**, 793–798 (1968).
40. H. Hu, X. Zhu, C. Wang, L. Zhang, X. Li, S. Lee, Z. Huang, R. Chen, Z. Chen, C. Wang, Y. Gu, Y. Chen, Y. Lei, T. Zhang, N. Kim, Y. Guo, Y. Teng, W. Zhou, Y. Li, A. Nomoto, S. Sternini, Q. Zhou, M. Pharr, F. L. di Scalea, S. Xu, Stretchable ultrasonic transducer arrays for three-dimensional imaging on complex surfaces. *Sci. Adv.* **4**, eaar3979 (2018).
41. P. N. T. Wells, Physical principles of ultrasonic diagnosis. *Med. Biol. Eng.* **8**, 219 (1970).
42. H. Yuk, S. Lin, C. Ma, M. Takaffoli, N. X. Fang, X. Zhao, Hydraulic hydrogel actuators and robots optically and sonically camouflaged in water. *Nat. Commun.* **8**, 14230 (2017).
43. T. Chiu, Z. Xiong, D. Parsons, M. R. Folkert, P. M. Medin, B. Hrycushko, Low-cost 3D print–based phantom fabrication to facilitate interstitial prostate brachytherapy training program. *Brachytherapy* **19**, 800–811 (2020).
44. N. Cardim, H. Dalen, J.-U. Voigt, A. Ionescu, S. Price, A. N. Neskovic, T. Edvardsen, M. Galderisi, R. Sicari, E. Donal, A. Stefanidis, V. Delgado, J. Zamorano, B. A. Popescu, The use of handheld ultrasound devices: A position statement of the European Association of Cardiovascular Imaging (2018 update). *Eur. Heart J. Cardiovasc. Imaging* **20**, 245–252 (2018).
